# Supplementary material for: Rewiring of a KNOXI regulatory network mediated by UFO underlies the compound leaf development in Medicago truncatula
Source: Nat Commun. 2024 Apr 6;15:2988. doi: 10.1038/s41467-024-47362-w (PMC10998843; doi:10.1038/s41467-024-47362-w)
Supplement: Supplementary file 1 — Supplementary Information [file 41467_2024_47362_MOESM1_ESM.pdf]

Supplementary Information

Rewiring of a KNOXI regulatory network mediated by UFO underlies the compound leaf development in *Medicago truncatula*

Lu et al.

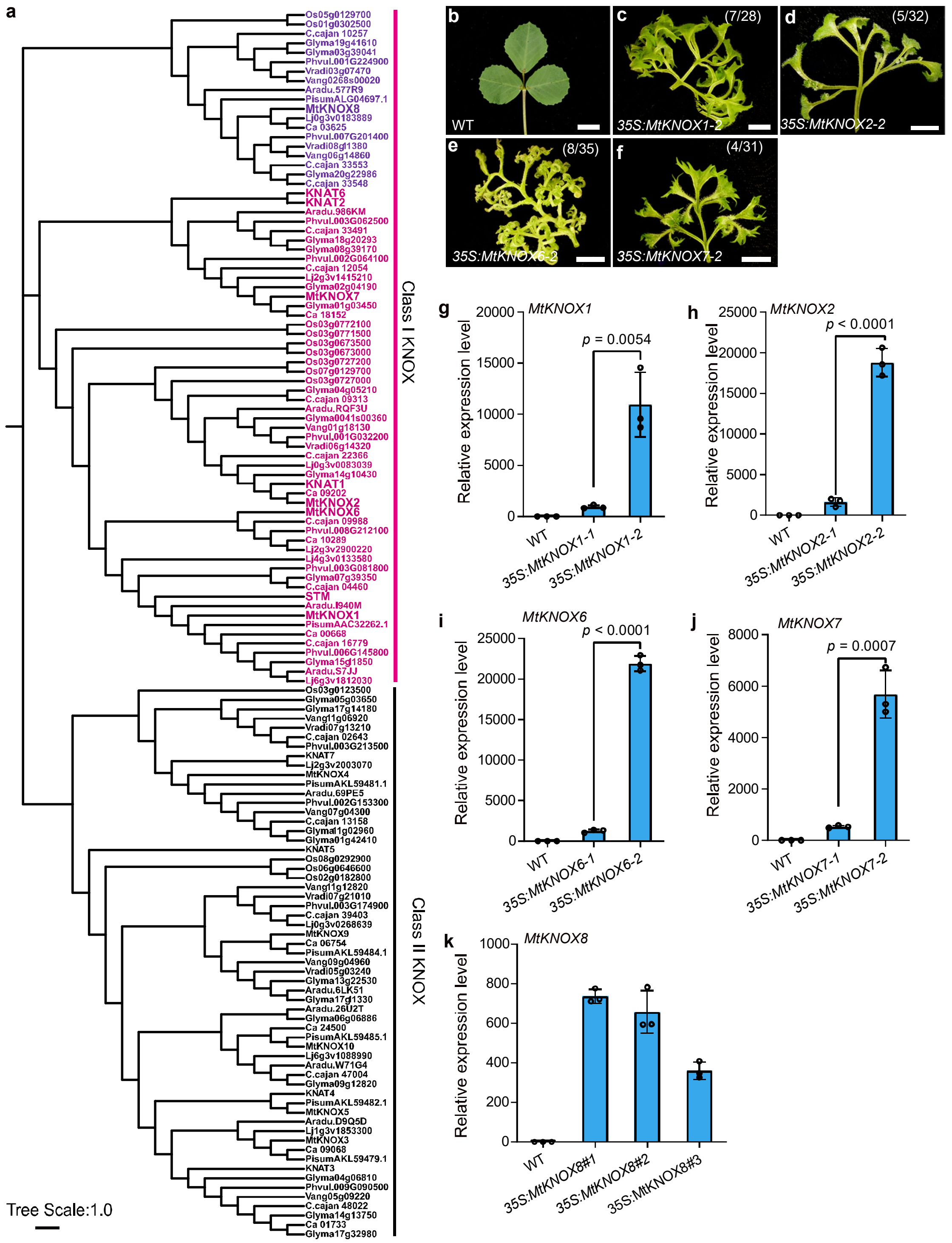

Supplementary Fig. 1. Ectopic MtKNOXI activity is sufficient for increasing leaf complexity. **a** Phylogenetic tree of KNOX proteins from several angiosperms (*A.thaliana*, *M. truncatula*, *C. cajan*, *G.max*, *L. japonicas*, *C. arietinum, P. vulgaris*, *A. duranensis*, *V. angularis*, *V.radiata*, *P. sativum* and *O.sativa*). **b** Mature leaf of WT. **c**-**f**, Representative leaves derived from transgenic plants overexpressing *35S:MtKNOX1* (**c**), *35S:MtKNOX2* (**d**), *35S:MtKNOX6* (**e**), and *35S:MtKNOX7* (**f**) with strong

phenotypes; (*w*/*o*) indicates that *w* in *o* total transgenic plants shows the displayed features. **g-k** Expression analysis of *MtKNOX1/2/6/7/8* in WT and transgenic plants by qRT–PCR. *MtUBIQUITIN* was used as the internal control. Data represent mean ± SD (n = 3 biological replicates), All above P values were calculated by unpaired two tailed t-test. Scale bar, 5 mm. Source data are provided as a Source Data file.

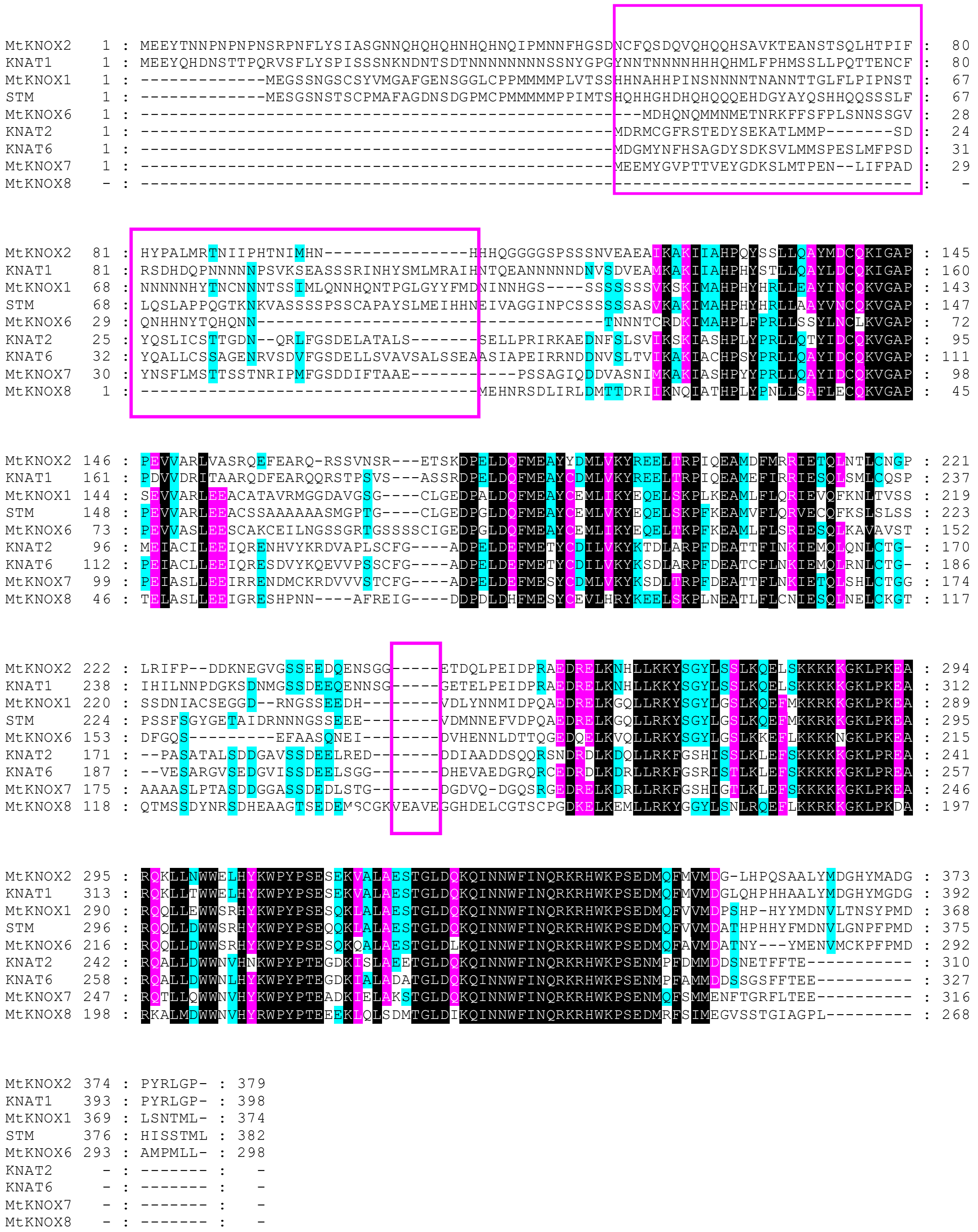

Supplementary Fig. 2. Multiple alignments of Class I KNOX proteins in *M. truncatula* and *A.thaliana.* The magenta boxes represent the main differences in the amino acid sequence between MtKNOX8 and other Class I KNOX proteins in *M. truncatula* and *A.thaliana* .

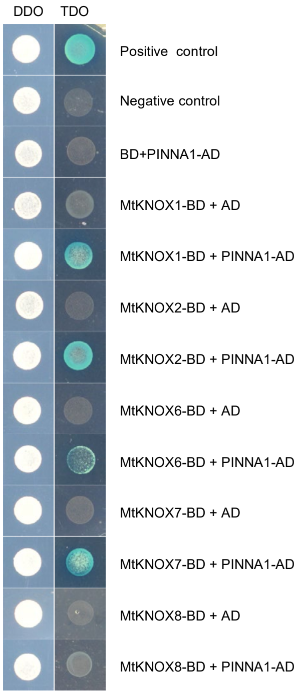

Supplementary Fig. 3. Yeast two-hybrid assay shows the interactions of PINNA1 and MtKNOXI. The yeast two-hybrid X-a-Gal filter assay showed that MtKNOX1/2/6/7 were able to interact with PINNA1. Transformed yeast cells were grown on DDO (SD/-Trp/-Leu) and TDO (SD/-Trp/-Leu/-His/ X-a-Gal). Similar results were obtained from three independent experiments.

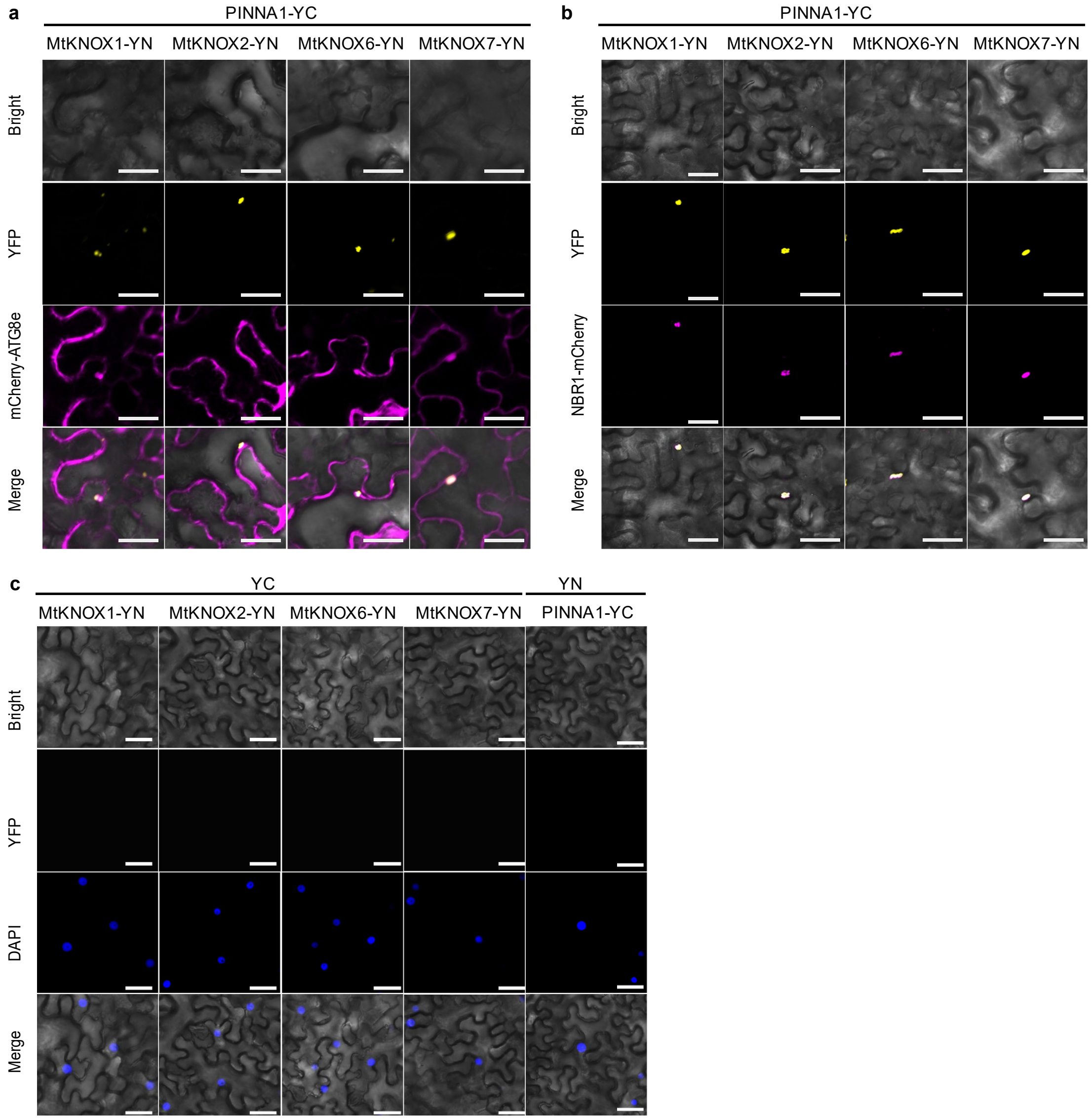

Supplementary Fig.4. BiFC complexes co-localized with mCherry-ATG8 or NBR1-mCherry in *N. benthamiana* leaves. a Co-localization of the BiFC signals and the autophagosome marker mCherry-ATG8e. **b** Co-localization of the BiFC signals and the autophagy receptor NBR1-mCherry. **c** Negative control of BiFC assay. YC was co-expressed with each MtKNOX1/2/6/7-YN, and YN was co-expressed with PINNA1-YC in *N. benthamiana* leaves. No interaction was found in any combination. Nuclei was stained by DAPI. All experiments were repeated three times biologically with similar results. Scale bar, 20 µm.

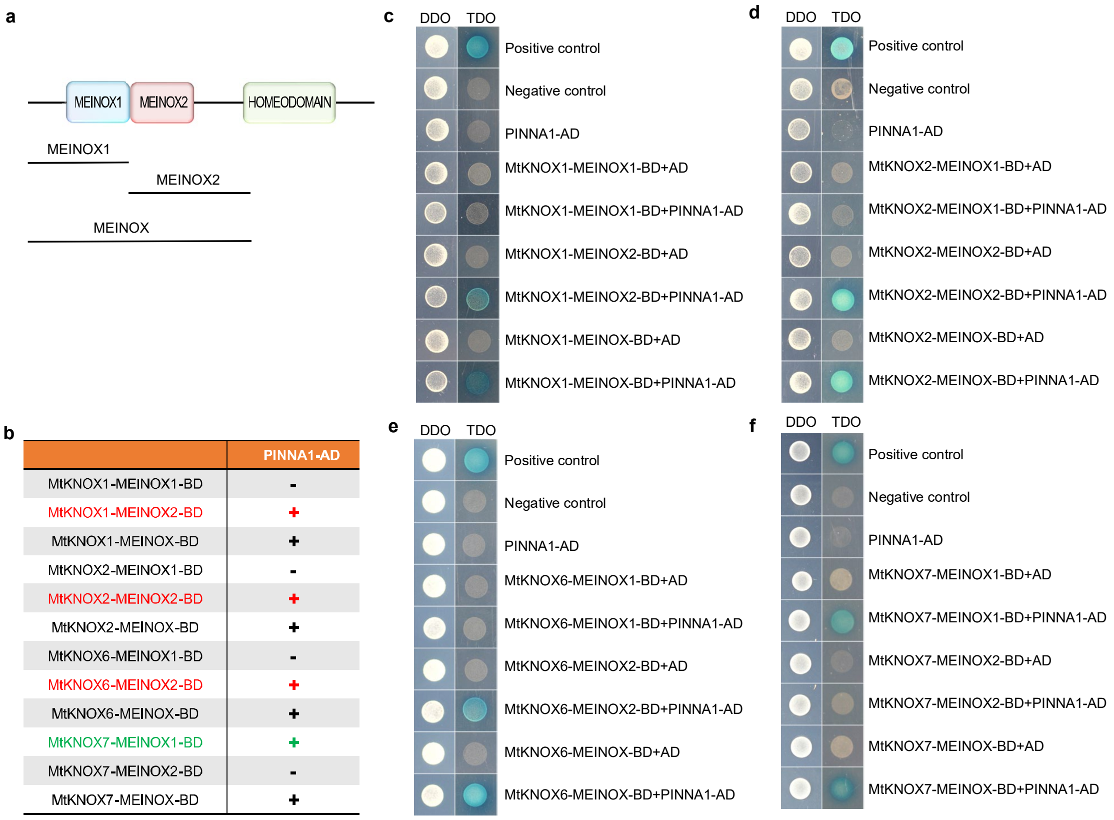

Supplementary Fig. 5. Yeast two-hybrid assay shows the interactions of PINNA1 and different fragments of the truncated MtKNOX1/2/6/7 proteins. a Schematic diagram of the truncated MtKNOX1/2/6/7 protein with three different fragments, MEINOX1, MEINOX2, and MEINOX domain. **b-f** The yeast two-hybrid assay analyzes the interactions of different fragments of the truncated MtKNOX1/2/6/7 proteins with PINNA1. Transformed yeast cells were grown on DDO (SD/-Trp/-Leu) and TDO (SD/-Trp/-Leu/-His/X-a-Gal). Similar results were obtained from three independent experiments.

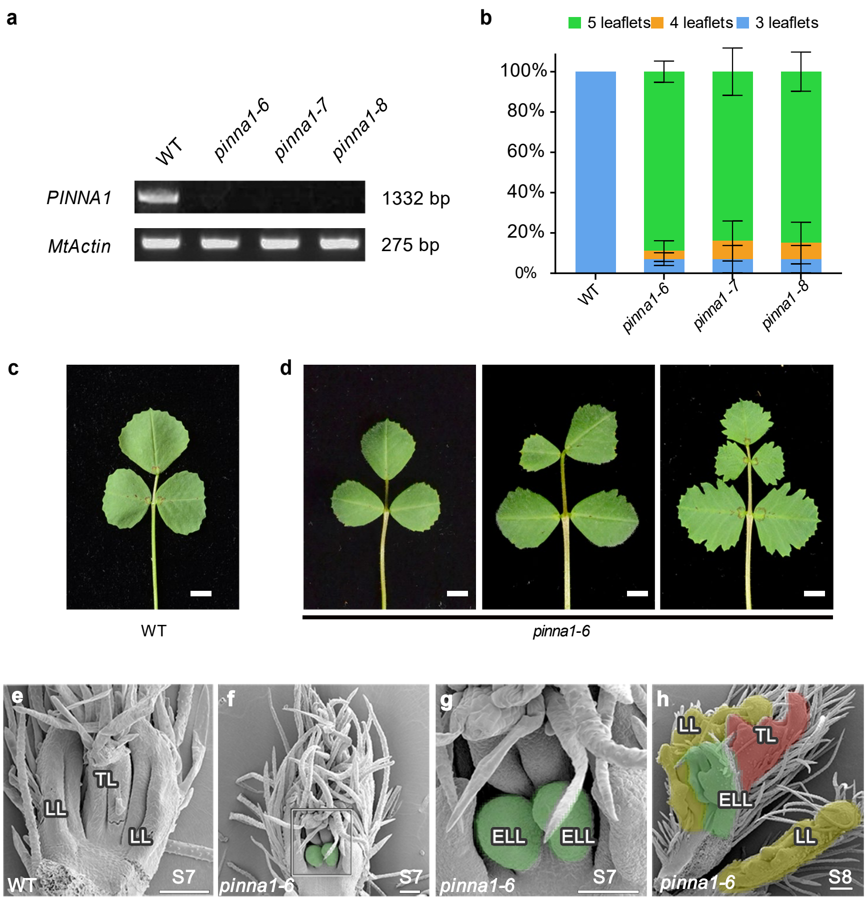

Supplementary Fig. 6. Molecular cloning and characterization of *PINNA1* in *M. truncatula*. **a** RT-PCR analysis of *PINNA1* transcripts in WT and *pinna1* mutants (*pinna1-6* to *pinna1-8*). *MtActin* was used as the loading control. Similar results were obtained from three independent experiments. **b** Proportion of leaves with different leaflet numbers in WT and *pinna1* mutants (*pinna1-6* to *pinna1-8*). 50-day-old plants were used for counting the leaflet numbers of adult leaves (*n*=10 leaves× two branches × five independent plants). Data represent mean ± SD. **c**, **d** Mature leaves of WT (**c**) and *pinna1-6* plants (**d**). **e**-**h** SEM analysis of leaf primordia in WT (**e**) and *pinna1-6* (**f**-**h**). (**g**) is high-magnification view of the white box of (**f**). Two ectopic lateral leaflets (ELL) are false-colored in green (**f**-**h**). Terminal leaflets (TL) are false-colored in pink (**h**) and the lateral leaflets (LL) are false-colored in yellow (**h**). Similar results were obtained from three biological replicates for each tissue or organ. Scale bar, 5 mm in (**c** and **d**), 20 µm in (**e**-**h**). Source data are provided as a Source Data file.

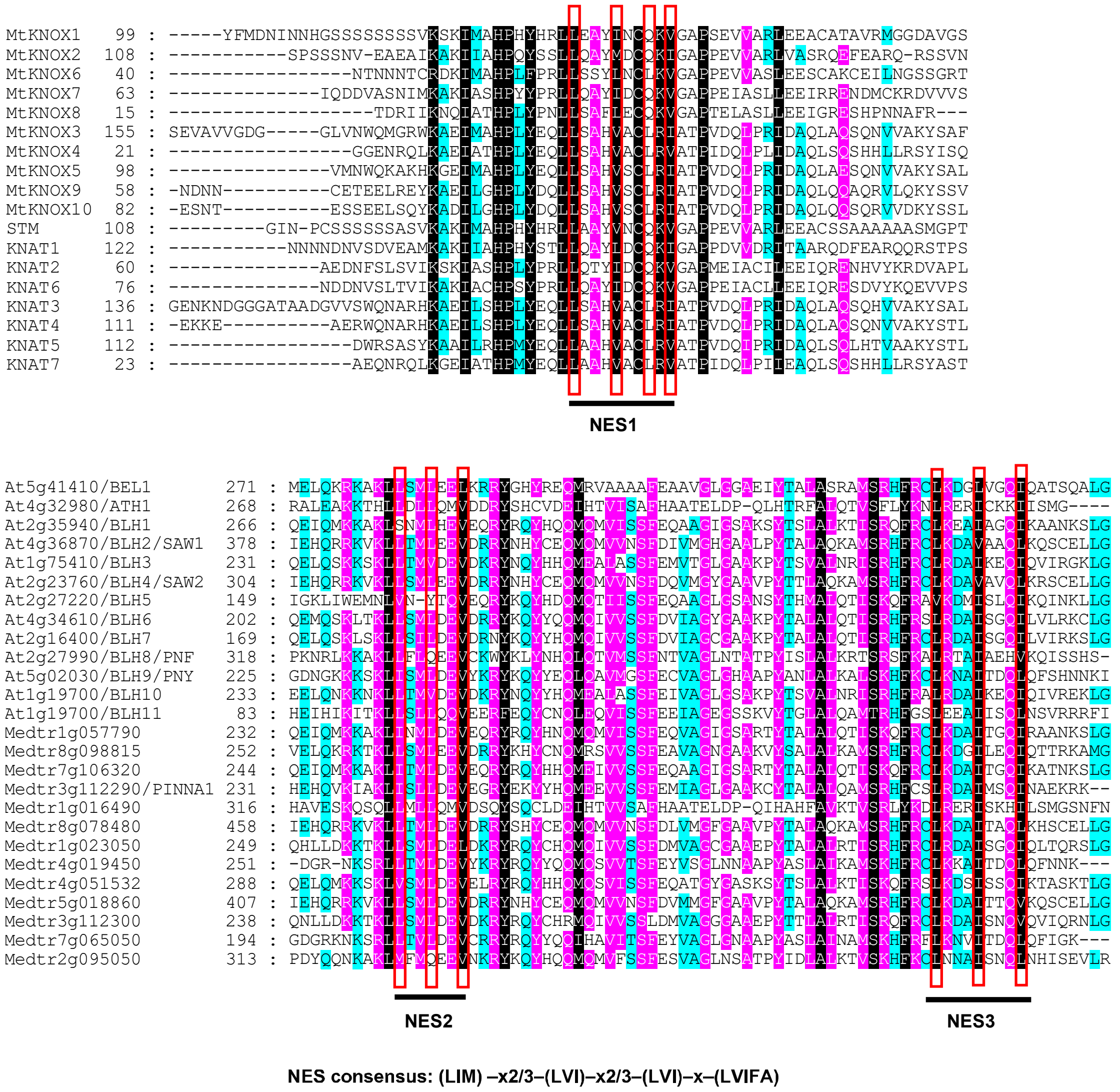

Supplementary Fig. 7. Conserved sequences of the nuclear export signal (NES) in the KNOX and BLH protein. Amino acid sequence alignment of the MEINOX1 domains or BELL domains from multiple KNOX proteins or BLH proteins in Arabidopsis and Medicago. The conserved sequences of the nuclear export signal (NES) in the MEINOX1 domains or BELL domains are indicated by red boxes. The NES consensus sequence is shown at the bottom.

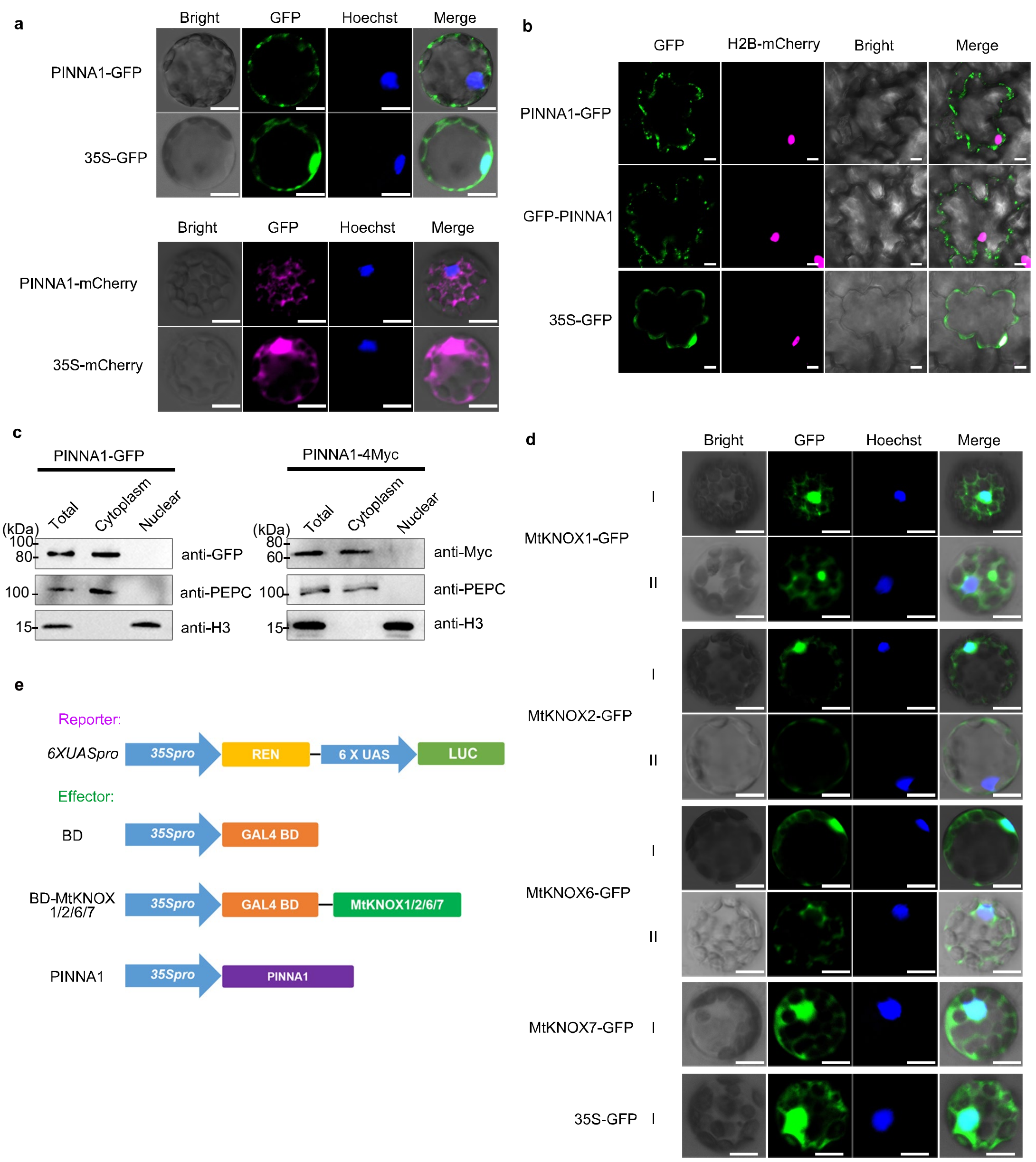

Supplementary Fig. 8. Subcellular localization of PINNA1 and MtKNOX1/2/6/7 protein in *M. truncatula*. **a** Subcellular localization of PINNA1-GFP, 35S-GFP, PINNA1-mCherry, and 35S-mCherry in *Medicago* mesophyll protoplast. **b** Subcellular localization of PINNA1-GFP, GFP-PINNA1, and 35S-GFP in *Medicago* leaf epidermal cells. H2B-mCherry was used as a nuclear marker. **c** Immunoblot analysis of protein expression in the nuclear and cytoplasmic fractions of *Medicago* mesophyll protoplasts transiently expressing PINNA1-GFP or PINNA1-4Myc alone. PEPC protein and Histone H3 (H3) were detected and used as cytoplasmic or nuclear markers. **d** Subcellular localization patterns of MtKNOX1/2/6/7-GFP in *Medicago* mesophyll protoplast. I, Type I (GFP signal was located in both cytoplasmic space and nucleus.). II, Type II (GFP signal was located only in the cytoplasmic space.). Hoechst 33342

(Hoechst) was used to stain the nuclei. **e** Schematic diagrams of the dual-luciferase reporter (DLR) assay system featuring different effectors. The *CaMV35S* promoter-driven REN (Renilla luciferase) served as an internal control, while the *6XUAS* promoter (*6XUASpro*)-driven LUC (Firefly luciferase) was used as the reporter. The effectors were GAL4 BD (BD), GAL4 BD-MtKNOX1/2/6/7 (BD-MtKNOX1/2/6/7), and PINNA1. All experiments were repeated three times biologically with similar results. Scale bar, 5 μm in (**a**, **b**, and **d**). Source data are provided as a Source Data file.

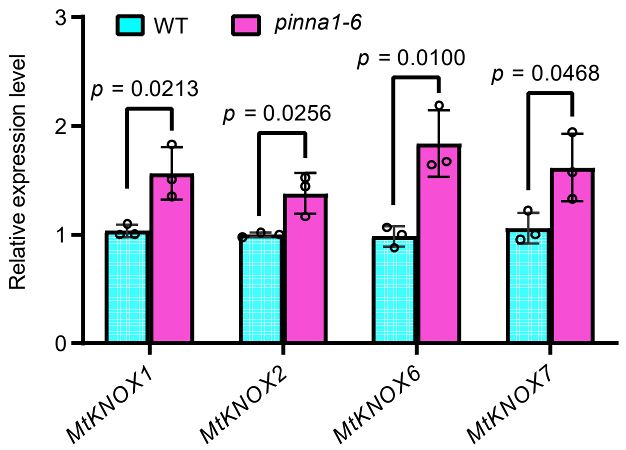

Supplementary Fig. 9. The expression levels of *MtKNOX1/2/6/7* in WT and *pinna1-6* plants. The expression levels of *MtKNOX1/2/6/7* in the shoot apices of WT and *pinna1-6* plants were determined by qRT-PCR. *MtUBIQUITIN* was used as the internal control. Data represent mean ± SD ($n = 3$ biological replicates), *P* values were calculated by unpaired two tailed *t*-test. Source data are provided as a Source Data file.

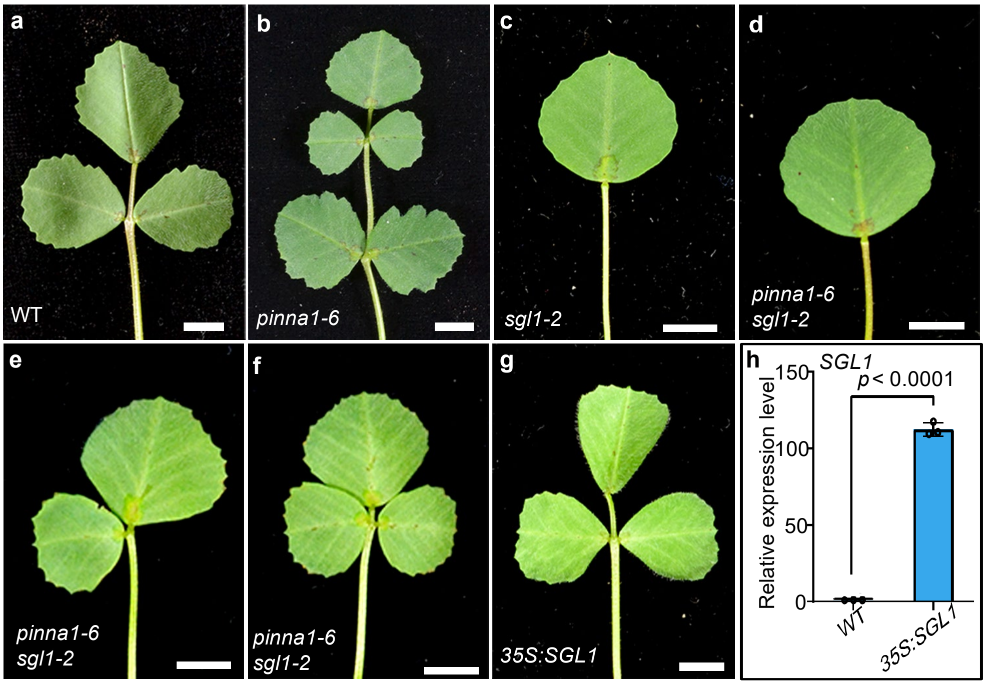

Supplementary Fig. 10. Representative leaves of some mutants. a-g Representative leaves of WT (**a**), *pinna1-6* (**b**), *sgl1-2* (**c**), *sgl1-2 pinna1-6* double mutants (**d-f**), and *35S:SGL1* transgenic plants (**g**). **h** The expression levels of *SGL1* in the shoot apices of WT and *35S:SGL1* were determined by qRT-PCR. *MtUBIQUITIN* was used as the internal control. Data represent mean ± SD (n = 3 biological replicates), P values were calculated by unpaired two tailed t-test. Scale bar, 5 mm on (**a-g**). Source data are provided as a Source Data file.

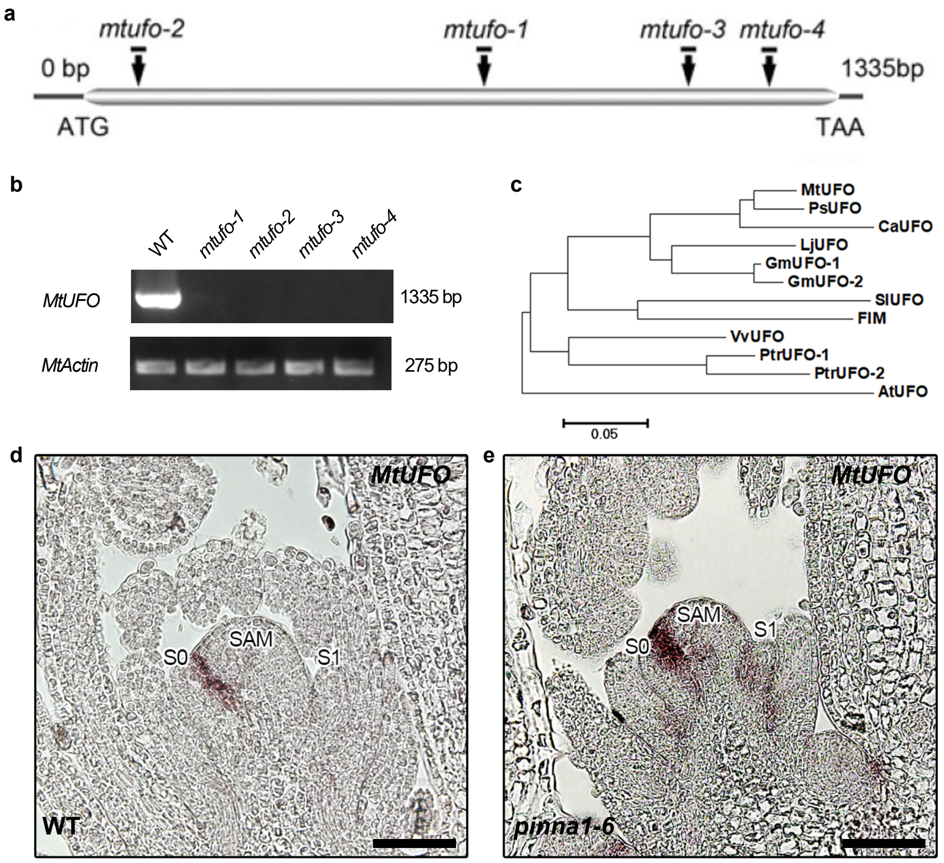

Supplementary Fig. 11. Molecular cloning of *MtUFO* in *M. truncatula*. a Schematic diagram of the *MtUFO* gene structure and *Tnt1* insertion positions in *mtufo* alleles. Boxes represent exons and lines represent introns. **b** RT-PCR analysis of the *MtUFO* transcripts in WT and *mtufo* mutants (*mtufo-1* to *mtufo-4*). **c** Phylogenetic tree of MtUFO and its orthologs in various species (*A. thaliana*, *M. truncatula*, *G. max*, *L. japonicas*, *E. guttata, C. arietinum*, *S. lycopersicum*, *P. trichocarpa*, *V. vinifera* and *P. sativum*). **d**, **e** RNA in situ hybridization analysis of *MtUFO* mRNA in the leaf primordia of WT (**d**) and *pinna1-6* (**e**). All experiments were repeated three times biologically with similar results. Scale bars, 50 μm in (**d** and **e**). Source data are provided as a Source Data file.

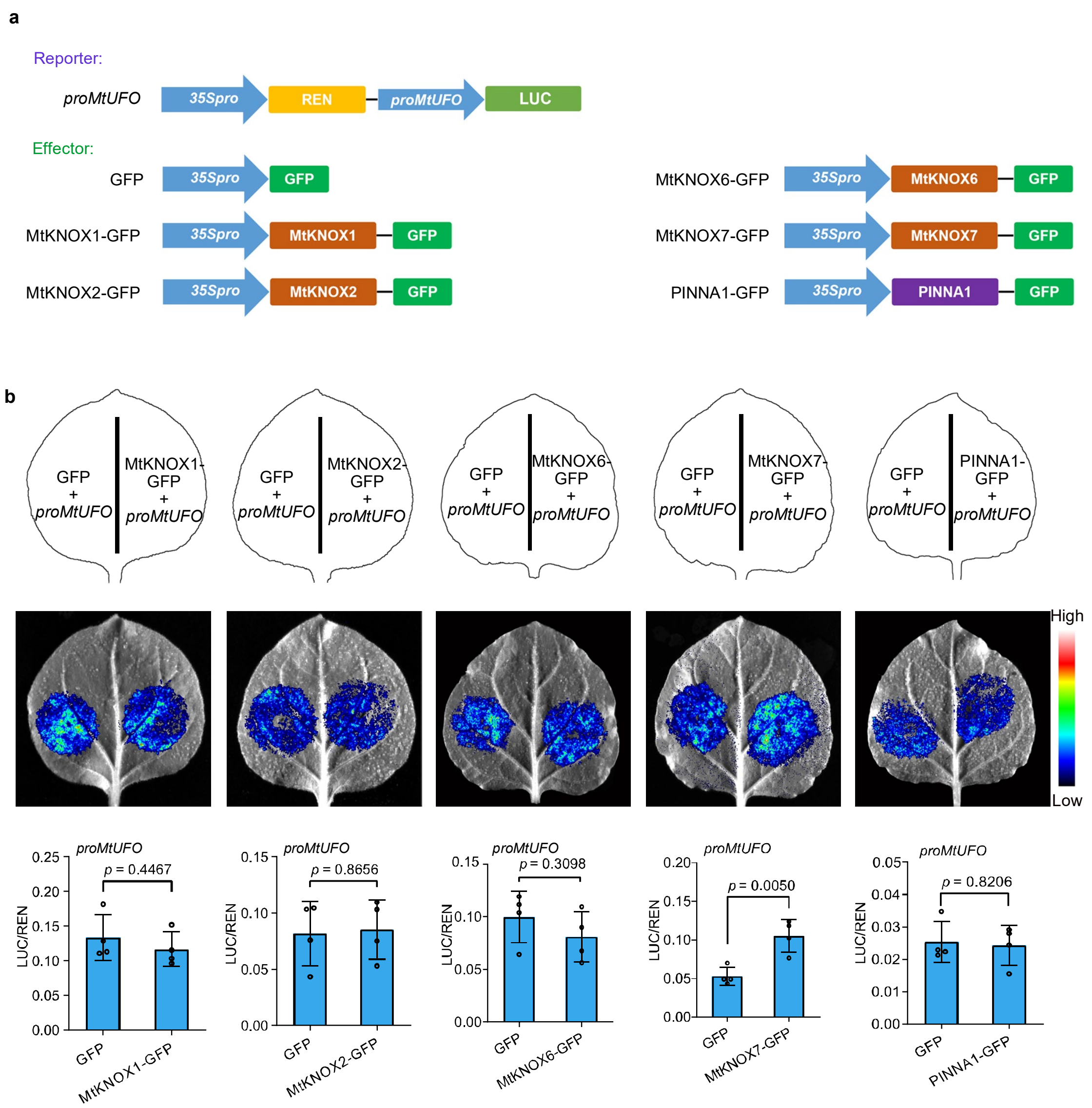

Supplementary Fig.12. MtKNOX7, instead of MtKNOX1/2/6, activates the *MtUFO* promoter. a Schematic representations of one reporter and six effectors for the DLR assay. The LUC was driven by the promoters of *MtUFO* (*pro MtUFO*), while the REN was under the control of the *CaMV35S* promoter (*35Spro*) as the internal control. **b** The top panels display the transient co-expression of the reporter and different effectors in tobacco leaves. The middle panels show color-coded LUC signal images. The bottom panels present the quantification of the ratio of LUC to REN activity. The DLR assay showed only MtKNOX7 activates the activity of the *MtUFO* promoter. Data represent mean ± SD ($n = 4$ biological replicates), P values were calculated by unpaired two tailed t-test. Source data are provided as a Source Data file.

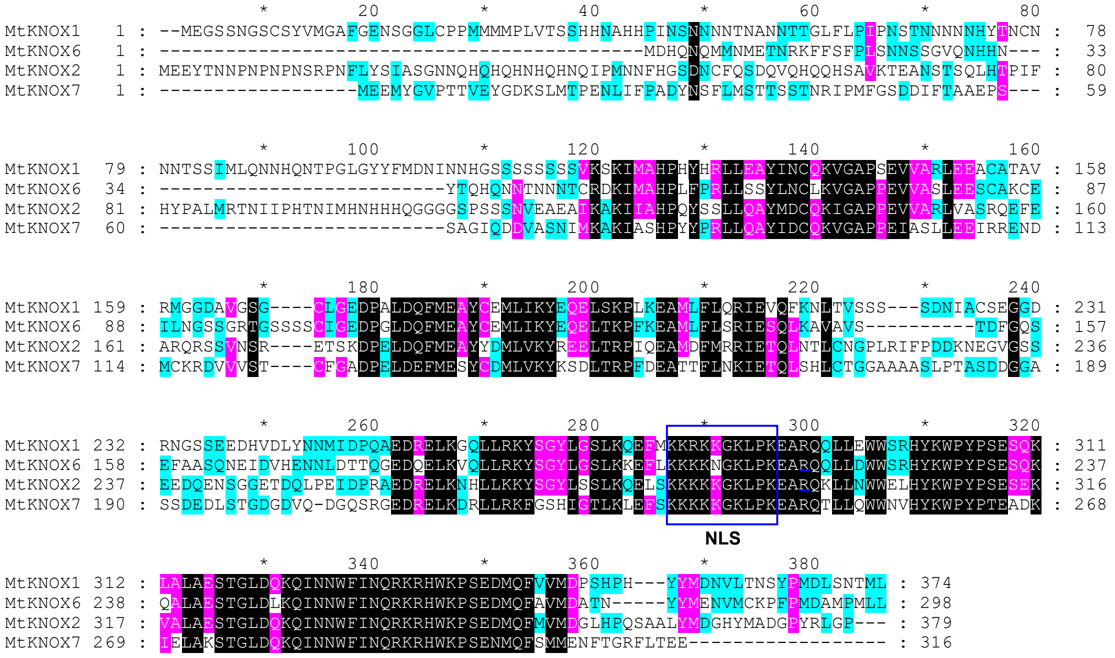

Supplementary Fig. 13. Conserved sequences of the nuclear localization signals (NLS) in the MtKNOX1/2/6/7 proteins. The blue box represents the NLS sequence in the MtKNOX1/2/6/7 proteins.

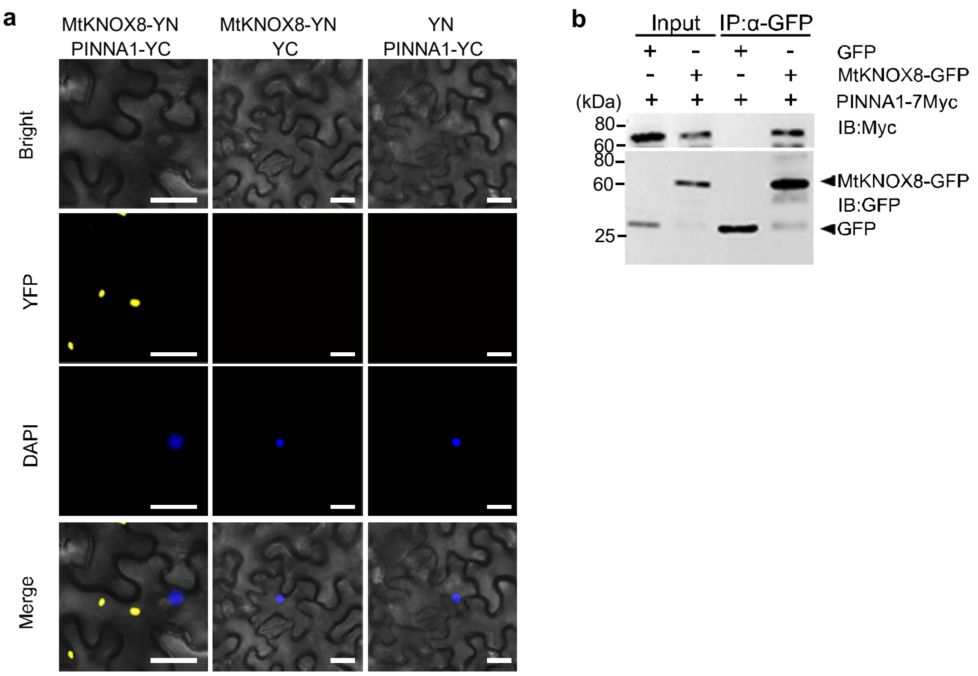

Supplementary Fig. 14. MtKNOX8 has a physical interaction with PINNA1 in *N. benthamiana* leaves. a BiFC assay showing the interaction between PINNA1 and MtKNOX8. DAPI was used to stain the nuclei. **b** Coimmunoprecipitation assay confirmed that PINNA1 interacts with MtKNOX8 in tobacco epidermal cells. Total proteins were immunoprecipitated using GFP-Trap beads, and the coimmunoprecipitated proteins were detected by the anti-Myc antibody. Three biological repeats were performed for the above experiments. Scale bars, 20 µm in (**a**). Source data are provided as a Source Data file.

Supplementary Table 1. List of *Medicago truncatula* mutants used in this study.

**Gene**	**Mutant allele**	**Location of *Tnt1***	**Flanking sequence of *Tnt1***
MtKNOX1	*Mtknox1-1*	1st exon	GGTGCTTT (*Tnt1*) TGGAGAAA
MtKNOX2	*mtknox2-1*	1st exon	GAAGCTAT (*Tnt1*) AAAAGCCA
MtKNOX6	*mtknox6-1*	3rd exon	AAAGTGCA (*Tnt1*) GCTTTTAC
MtKNOX7	*mtknox7-1*	1st exon	CGGATCCG (*Tnt1*) ACGATATC
MtKNOX7	*mtknox7-2*	1st exon	TGAATTTA (*Tnt1*) TGGTCCCC
PINNA1	*pinna1-6*	1st exon	GTCTTATG (*Tnt1*) ATCAATCC
PINNA1	*pinna1-7*	1st exon	CTGGTCAA (*Tnt1*) GATCAGAC
PINNA1	*pinna1-8*	3rd exon	TTTAACCA (*Tnt1*) AAAACCAA
MtUFO	*mtufo-1*	1st exon	ATCACCTT (*Tnt1*) ATGCAGTT
SGL1	*sgl1-2*	1st exon	TGGGATCT (*Tnt1*) TCTTGTTG
