# Supplementary material for: Rewiring of a KNOXI regulatory network mediated by UFO underlies the compound leaf development in Medicago truncatula
Source: Nat Commun. 2024 Apr 6;15:2988. doi: 10.1038/s41467-024-47362-w (PMC10998843; doi:10.1038/s41467-024-47362-w)
Supplement: Supplementary file 3 — Description of additional supplementary files [file 41467_2024_47362_MOESM3_ESM.pdf]

<u>Description of Additional Supplementary Files</u>

File Name: Supplementary Data 1.

Description: PCR primer sequences used in this study.

File Name: Supplementary Data 2.

Description: Alignment of KNOX homologs from different legume species in Fig.1a (To open this file, you need to rename it as a .aln file and then open it using ClustalX 2.1).

File Name: Supplementary Data 3.

Description: Alignment of KNOX homologs from different legume species and rice in Supplementary Fig.1a (To open this file, you need to rename it as a .aln file and then open it using ClustalX 2.1).

File Name: Supplementary Data 4.

Description: Alignment of MtUFO orthologs from different species in Supplementary Fig.11c (To open this file, you need to rename it as a .aln file and then open it using ClustalX 2.1)
